# Molecular detection and characterization of SARS-CoV-2 in cats and dogs of positive owners during the first COVID-19 wave in Brazil

**DOI:** 10.1038/s41598-023-41285-0

**Published:** 2023-09-02

**Authors:** Juliana Arena Galhardo, David Soeiro Barbosa, Louise Bach Kmetiuk, Otávio Valério de Carvalho, Ana Izabel Passarella Teixeira, Paula Luize Camargos Fonseca, Luiza Campos Guerra de Araújo e Santos, Daniel Costa Queiroz, João Victor Oliveira Miranda, Aluisio Pereira da Silva Filho, Anisleidy Pérez Castillo, Ricardo Nascimento Araujo, Julia Angelica Gonçalves da Silveira, Luiz Eduardo Ristow, Daniel Friguglietti Brandespim, Christina Pettan-Brewer, Ana Marcia de Sá Guimarães, Valéria Dutra, Helio Autran de Morais, Andrea Pires dos Santos, Rafael Garabet Agopian, Renato Santana de Aguiar, Alexander Welker Biondo

**Affiliations:** 1 College of Veterinary Medicine and Animal Science, Federal University of Mato Grosso do Sul (UFMS), Campo Grande, MS Brazil; 2 Department of Parasitology, Institute of Biological Sciences, Federal University of Minas Gerais (UFMG), Belo Horizonte, MG Brazil; 3 Graduate College of Cellular and Molecular Biology, Federal University of Paraná (UFPR), Curitiba, PR Brazil; 4 College of Veterinary Medicine, Campus Paranaíba, Federal University of Mato Grosso do Sul, Paranaíba, Mato Grosso do Sul Brazil; 5 Department of Genetics, Ecology and Evolution, Institute of Biological Sciences, Federal University of Minas Gerais, Belo Horizonte, Minas Gerais Brazil; 6 Department of Veterinary Medicine, College of Veterinary Medicine, Federal Rural University of Pernambuco (UFRPE), Recife, PE Brazil; 7 Department of Comparative Medicine, School of Medicine, University of Washington, Seattle, WA USA; 8 Department of Microbiology, Institute of Biomedical Sciences, University of São Paulo (USP), São Paulo, SP Brazil; 9 Department of Veterinary Medicine, College of Veterinary Medicine, Federal University of Mato Grosso (UFMT), Cuiabá, MT Brazil; 10 Department of Clinical Sciences, Oregon State University, Corvallis, OR USA; 11 Department of Comparative Pathobiology, College of Veterinary Medicine, Purdue University, West Lafayette, IN USA; 12 Department of Veterinary Medicine, University of Santo Amaro, São Paulo, Brazil; 13 Department of Veterinary Medicine, Federal University of Paraná (UFPR), Curitiba, PR Brazil

**Keywords:** Molecular biology, Genomic analysis

## Abstract

Despite previous reports of SARS-CoV-2 infection in dogs and cats worldwide, the type of swab sample used for its detection through RT-qPCR needs to be better compared and described. Accordingly, as part of a multicenter study in Brazil, the aim of the present study was to assess which rectal or oropharyngeal swabs would be more appropriate for detecting SARS-CoV-2 in cats and dogs, through viral load comparison. Pets of owners diagnosed with COVID-19 in the last 7 days were eligible. A total of 148 animals from four of the five Brazilian geographical regions were analyzed, among which 10/48 cats (20.83%) and 11/100 dogs (11.00%) were positive. The results suggested that oropharyngeal swabs should be considered for SARS-CoV-2 detection, particularly in cats, due to the higher cDNA viral load. Also, the genomic results showed similarities between SARS-CoV-2 animal variants and human variants that were circulating at the time of sampling, thus corroborating the existence of zooanthroponotic transmission. In conclusion, the present study highlighted the importance of SARS-CoV-2 monitoring among cats and dogs, as virus modification may indicate the possibility of mutations in animals and spillover back to owners. Thus, positive individuals should always self-isolate from their pets during COVID-19, to prevent trans-species transmission and mutation.

## Introduction

SARS-CoV-2 (severe acute respiratory syndrome coronavirus 2) is the etiological agent responsible for COVID-19 (coronavirus disease). COVID-19 started in Wuhan, China, and spread widely, leading to declaration of a pandemic by the World Health Organization (WHO)^1,2^. Despite SARS-CoV-2 has been identified in several animal species, transmission from animals to people has been reportedly suggested only in domestic cats (*Felis catus*)^3^, Syrian hamsters (*Mesocricetus auratus*)^4^, American minks^5^ and white-tailed deer^6^. Among pet animals, domestic cats and Syrian hamsters are considered to be the species most susceptible to SARS-CoV-2^7–9^, whereas dogs (*Canis lupus familiaris*) appear to be more resistant to infection and disease^10–12^.

The SARS-CoV-2 variants identified in cats and dogs are genetically similar to the ones that were circulating in humans at the same time, thus suggesting that transmission from people to domestic animals (zooanthroponotic transmission) is a common and ongoing occurrence^13,14^. Moreover, the epidemiological role that those animals could play in viral transmission to humans has also been studied. Cats herein appeared to play a minor role in SARS-CoV-2 transmission to humans due to low viral circulation and shedding among studied cats, along with previously observed viral shedding period of around 5 days^15^. However, such findings remain controversial, since infected cats may shed high loads of viable virus, enough to infect veterinarians, as already reported^3^.

Despite many reports, the type of sampling used (oral, rectal or oropharyngeal swabs) to obtain SARS-CoV-2 genetic material for RT-qPCR (reverse transcriptase quantitative polymerase chain reaction) has varied among studies. Although each method can identify virus genes from biological samples^16–19^, and viral load may vary between tissues, such techniques were not fully established or validated in affected animals^20^. Accordingly, the aim of the present study was to assess which rectal or oropharyngeal swab would be more appropriate for detecting SARS-CoV-2 in cats and dogs, through viral load comparison. Genomic sequencing was also evaluated to compare the samples isolated with concurrently circulating variants.

## Results

### Sample description

**Figure 1:**
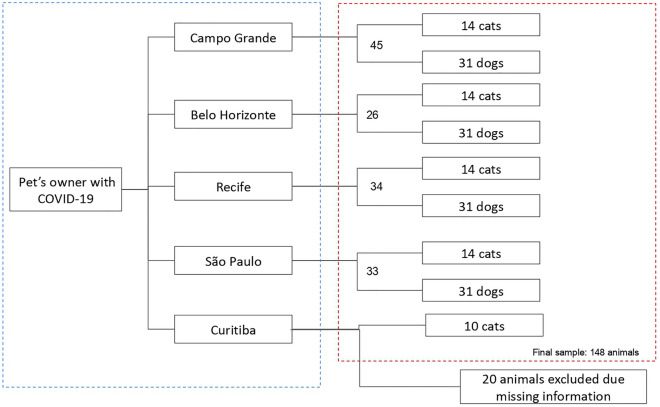
Recruitment flow and number of animals included in the study per city, Brazil.

A total of 148 animals were included in this study: 48 cats and 100 dogs, as shown in Fig. 1. The 148 companion animals herein were from 96 visited households, of which 9 households had more than one animal included in the survey. Among the Brazilian geographical regions, 33.1% (19/148) were from the southeastern region, 30.4% (45/148) central-western region, 22.9% (34/148) northeastern region and 6.7% (10/148) southern region.

The animals consisted of 60.42% female cats (29/48) and 46.46% female dogs (46/99; sex information missing in one case); 54% (54/100) of the dogs had been neutered, among which 44.44% (24/54) were female; and 89.60% (43/48) of the cats had been castrated, among which 62.80% (27/43) were female.

The median age of the dogs was 72 months (average 74.09, interquartiles 30.75–100.5). The median age of the cats was 36 months (average 57.33; interquartiles 12.00–95.00).

Most of the animals did not have a defined breed according to their owners; 89.60% (43/48) were cats without a defined breed, and 45.45% (45/98; two cases of missing information) were dogs without a defined breed. Among the cat owners, 12.5% (6/48) reported that their animal had a comorbidity, and the one most reported was chronic kidney disease, in three cats. Among the dog owners, 30.00% (30/100) reported that their animal had a comorbidity: leishmaniasis was reported in 26.66% (8/30) of the dogs. In the physical examination, 20.83% (10/48) of the cats and 18.00% (18/100) of the dogs were considered symptomatic for respiratory illness. Presence of clinical signs was assessed in all companion animals regardless of positivity, with 40.00% (4/10) of symptomatic cats and 11.11% (2/18) of symptomatic dogs positive for SARS-CoV-2 (Supplementary Table S1). The inclusion criterium was the timeline of positive COVID-19 owners (and not their animals), as the timeline of animal symptoms was not available at the time.

Regarding detection of SARS-CoV-2, 14.19% (95% CI 9.47–20.72; 21/148) of animals tested positive by rectal and/or oropharyngeal swabs. Analysis according to species stratum has shown 20.83% (95% CI 11.73–34.26; 10/48) positive cats and 11.00% (95% CI 6.25–18.63; 11/100) positive dogs. Only six/21 positive animals were identified as symptomatic. A total of 25 RT-qPCR results were positive, indicating 21 positive animals, with 2 positive animals in both swabs. In summary, 18 oropharyngeal swabs were positive for SARS-CoV-2 gene detection, along with two positive rectal swabs, and two animals with both positive rectal and oropharyngeal swabs.

**Figure 2:**
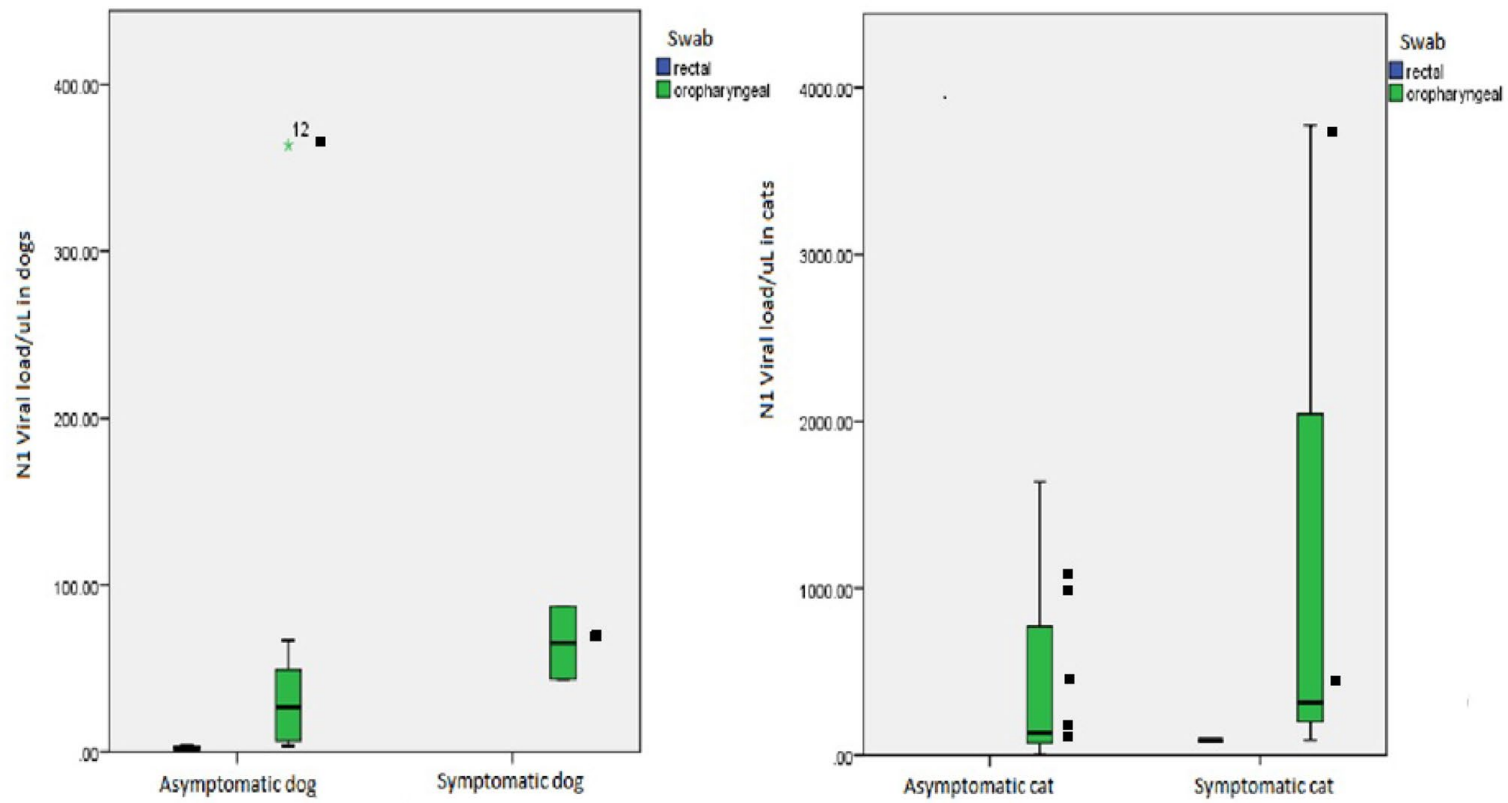
RNA viral load of the SARS-CoV-2 N1 gene in symptomatic and asymptomatic dogs and cats. *Black square indicates the samples that went to genetic sequencing.

We recovered SARS-CoV-2 RNA from both symptomatic and asymptomatic animals. Viral RNA quantification was higher in oropharyngeal swabs (mean 435.74 copies/µL, median 87.26 copies/µL, interquartile range 11.36–363.51 copies/µL) than in rectal swabs (mean 24.03 copies/µL, median 2.94 copies/µL, interquartile range 1.24–67.93 copies/µL), with a p-value of 0.035 in the Mann–Whitney test. Also, more viral RNA was recovered from symptomatic animals than from asymptomatic animals, as shown in Fig. 2.

**Figure 3:**
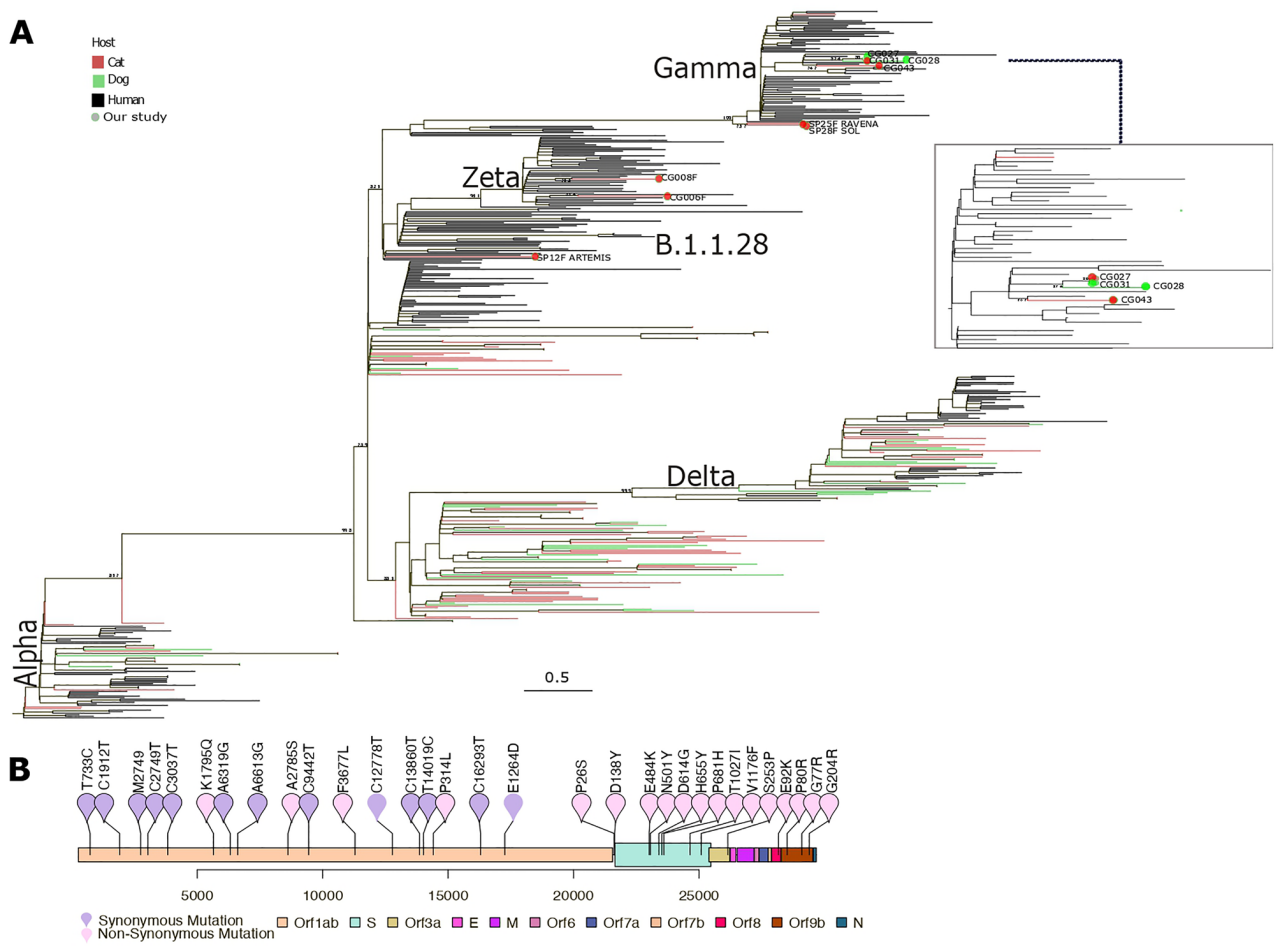
Maximum likelihood phylogenetic tree. Dataset was constructed based on 456 public sequences from the GISAID database, and nine sequences generated in the present study. Circle Tip indicates genomes generated by our study. Red circle tips indicate the SARS-CoV-2 genome isolated from cats. Green circle tips indicate the SARS-CoV-2 genome isolated from dogs. The color of each branch indicates the host species from which the genome was isolated, cats in red, dogs in green, and humans in black. (**B**) Schematic representation of the samples CG027F and CG031F. Both genomes present the same mutation profile. Pink dots represents non-synonymous mutations, while purple dots represent synonymous mutations.

Nine samples were sequenced. The average genome coverage was 93.22% with a mean depth of 13,639.61x. The sequencing metrics and lineage classification of the genomes are available in Supplementary Table S1. One sample was classified using the Pangolin tool as B.1.1.28 lineage, two samples as Zeta and six samples as VOC Gamma (4 as P.1 and 2 as P.1.7). The phylogenetic analyses (Fig. 3) confirmed the classification generated by Pangolin. Two cat samples were classified as VOC Gamma (SP25F RAVENA and SP28F SOL), and one dog (CG027C) and one cat (CG031F) showed high similarity. The samples SP25F RAVENA and SP28F SOL presented a genomic difference between them. The sample SP25F RAVENA had two additional mutations (C843A and C2773T). High similarity was found in samples from a dog (CG027C) and a cat (CG031F) in the same genetic cluster (Fig. 3, Supplementary Table S2). These two samples were classified as sublineage P.1.7 and presented the same mutation profile (Supplementary Table S2; https://github.com/LBI-lab/PETCOVID). Moreover, the genetic distance between sequences was 0.000002, indicating that these samples were very close to each other (Supplementary Table S2). A genomic difference was also found between samples SP25F RAVENA and SP28F SOL, with sample SP25F RAVENA presenting two additional mutations ORF1a: P193H (C843A) and ORF1a: C2773T.

## Discussion

Detection of SARS-CoV-2 in domestic animals whose owners are positive for SARS-CoV-2 has already been studied. In a study carried out in France, no virus was detected in the 21 animals evaluated, even with positive owners^17^. Similar studies were also conducted in Brazil; in one study, 96 animals tested were all negative for SARS-CoV-2^21^. In another study, two cats and six dogs tested positive for SARS-CoV-2 through nasopharyngeal swab detection^10^. Two more studies showed positivity for SARS-CoV-2; in one, detected in two asymptomatic cats using nasopharyngeal swabs^18^; and in the other, detected in five dogs also using nasopharyngeal swabs^18^. The present study showed positivity in 21 of the 148 animals evaluated: 10/48 cats and 11/100 dogs. These results corroborate those of other studies on detection of SARS-CoV-2 in domestic animals, with greater positivity among cats than among dogs, whose owners were SARS-CoV-2-positive^22,23^. The difference in the proportion of positivity among studies may probably also be due to methodological differences related to how the animals were selected. In the present study, companion animals were obtained from owners with diagnosed COVID-19.

The present study also showed that most positive animals were considered asymptomatic, in line with the literature^24,25^. Although SARS-CoV-2-related deaths in animals have been reported^26^, no lethal cases due to SARS-CoV-2 infection were observed or reported in the present study.

The high rate of detection through oropharyngeal swabs was similar to results from other studies^10,18,27^ and possibly can be considered to be the recommended method for detection of SARS-CoV-2 in oropharyngeal swab samples from cats, because of the higher detection of viral genome. Also, the genomic results showed presence of variants similar to the variants that were predominantly infecting humans at the time, which strengthens the scientific evidence for zooanthroponotic transmission^13,14^.

Genomic similarity was observed between cat samples SP25F RAVENA and SP28F SOL, and between dog sample CG027C and cat sample CG031F. Cat samples SP25F RAVENA and SP28F SOL lived in the same household; in another non-related household lived the dog sample CG027C and cat sample CG031F. In addition, the sample SP25F RAVENA presented a genomic polymorphism when compared to SP28F SOL. The sample SP25F RAVENA had two additional mutations (C843A and C2773T), suggesting virus intra-host microevolution, as previously described^28^. This variation can be observed in the phylogenetic tree of the present study (Fig. 3) since the branch lengths differ between the SP25F RAVENA and SP28F SOL samples. These findings have highlighted the importance of sequencing human and companion animal samples in order to pinpoint circulating lineages of SARS-CoV-2 in human and animal populations. As intra-household contact between owners and their pets have increased during COVID quarantine periods^29^, such human-animal close and continuous interactions may have contributed to viral evolution and new mutations, as shown in the present study.

Despite not having enough evidence to discuss the possibility of animal-to-human SARS-CoV-2 transmission, highlighted by the high yield of cDNA recovered from these animals, cannot be further discussed because there was insufficient evidence. This result showed that infected mammals could possibly have higher viral shedding than previously described^30^.

Limitations of the study herein were similar to other descriptive cross-sectional on field studies, including low time accuracy, without possibility of causation and also susceptible to survey bias due to low response and misclassification. Thus, positive individuals should always self-isolate from their pets during COVID-19, to prevent trans-species transmission and mutation. These recommendations are in line with what is recommended by international health organizations such as the CDC and WHO^31,32^.

## Methods

### Study design

This was an observational study conducted between October 2020 and June 2021 in five large metropolitan areas in Brazil, belonging to four of the five different Brazilian geographical regions (Recife, Belo Horizonte, São Paulo, Campo Grande and Curitiba). Dogs and cats whose owners tested positive for SARS-CoV-2 by means of RT-qPCR in the previous 7 days were eligible to enter the study.

### Ethical considerations

The authors confirm that the procedures complied with national and Brazilian legislation. The study was approved by the Ethics Committee on Animal Use of the Federal Rural University of Pernambuco (protocol number 4879280420, ID 000256) and by the Ethics Committee for Research on Human Beings of the Federal University of Mato Grosso do Sul (CEP–UFMS no. 4.470.448), as part of the multicenter study Pet-COVID-19 (CNPq no. 402341/2020-1). Before participating in the present study, each dog and cat owner gave informed written consent for the study results to be used. All the procedures were designed to reduce animal suffering, and all owners were informed about the possible risks and how to protect the research team from SARS-CoV-2 infection through adequate use of personal protection equipment. Reporting of results follows the recommendations of the ARRIVE guidelines.

### Study population and area

This research was conducted as a component of the multicenter Pet-COVID-19 study (CNPq no. 402341/2020–1), which was carried out in five cities that serve as the capitals of their states: Campo Grande (Mato Grosso do Sul), Belo Horizonte (Minas Gerais), Recife (Pernambuco), Sao Paulo (Sao Paulo) and Curitiba (Paraná).

Cats and dogs from households where people tested positive for SARS-CoV-2 by RT-qPCR, up to prior seven days maximum, were eligible and included in the study. Aggressive animals from which samples could not be safely collected were excluded from the study.

### Recruitment strategy

Recruitment was sought through the mass media. Prospective participants in the metropolitan areas of the cities of this study were instructed to contact the investigators to schedule a home visit for animal sample collections, over the period from October 2020 to June 2021. The outcome considered for this study was testing positive through RT-qPCR.

### Animal clinical evaluation

The animals underwent a physical examination prior to sample collection. Dogs and cats with respiratory or gastrointestinal signs were considered symptomatic, whereas healthy animals and animals with signs related to other organ systems were included in the non-symptomatic group. The team members were given adequate personal protection equipment (i.e. gloves, masks and 70% alcohol) and, during the visitation, they performed a physical evaluation on the animal and collected rectal and oropharyngeal swabs. The swabs were placed in tubes, refrigerated and sent to the laboratory facility.

In the physical examination, all animal body systems were evaluated through palpation, visualization, percussion, and auscultation. Animals presenting relevant respiratory and/or digestive clinical signs were considered symptomatic cases.

### Swab collection

The animals were sampled via oropharyngeal and rectal swabs by a certified veterinarian after physical restraint, if necessary. One rectal swab and one oropharyngeal swab were collected from each animal in a household with a SARS-COV-2-positive owner. These were placed in separate tubes for transportation.

The rectal swab was collected by introducing the swab into the animal’s rectum, followed by rotational movements in the rectal mucosa to collect cells. The oropharyngeal swab was collected by introducing the swab into the oral cavity until it reached the oropharynx, followed by rotation movements to collect cells and secretions. The tubes were kept at refrigeration temperatures (3–5 °C) until their arrival at the laboratory facility.

### RT-qPCR analysis

The RT-qPCR analysis was performed at TECSA Laboratories (Belo Horizonte, Brazil), and were carried out as previously described^18^. When necessary, confirmatory tests were performed at the Integrative Biology Laboratory/Institute of Biological Sciences at the Federal University of Minas Gerais. First, the swab tubes were vortexed for 30 s; then the RNA was isolated using a magnetic bead-based nucleic acid extraction in 500 µL obtained from the supernatant of the sample. Total RNA from the samples was extracted using a commercial kit (Maxwell® RSC simplyRNA Tissue Kit, Promega Corp., Madison, WI, USA) and an automated platform (Maxwell® RSC 48, Promega Corp., Madison, WI, USA), in accordance with the manufacturer’s instructions.

SARS-CoV-2 RT-qPCR tests were performed using two commercial kits: GoTaq® 1-Step RT (Promega Corp., Madison, WI, USA) and 2019-nCoV (Integrated DNA Technologies—IDT, Coralville, IA, USA). These targeted two regions from the nucleocapsid (N1 and N2) gene for specific detection of SARS-CoV-2. RT-qPCR was carried out in a commercial thermocycler (QuantStudio™ 1–96-well 0.2 mL block; Thermo Fisher Scientific, Waltham, MA, USA). Samples were considered positive when two viral target cycle threshold values (Ct-values) were below 40. Samples from which only one target was amplified were considered inconclusive. In addition, individual Ct curves were presented (Supplementary Table S3).

Quantification of SARS-CoV-2 RNA was performed using the same reaction. A commercial control (2019-nCoV Positive Control; Integrated DNA Technologies—IDT, Coralville, IA, USA) was used to provide a standard curve (five dilution points), and the feline β-actin and canine β-actin genes were used as internal control genes in testing on each species, respectively.

### Data collection

All the research teams in the different cities collected information, and the data were tabulated and integrated for all centers. The information collected encompassed the visitation data, biological information about the pet and physical evaluation, along with the RT-qPCR results with Ct curves and cDNA quantification. Animals with incomplete physical evaluations or missing data were excluded.

### Genome sequencing and phylogenetic analysis

The analysis was performed at the Integrative Biology Laboratory in the Institute of Biological Sciences at the Federal University of Minas Gerais. Nine samples with Ct < 30 were subjected to SARS-CoV-2 whole-genome sequencing. Sequencing libraries were prepared using the QIAseq FX DNA Library Prep kit (QIAGEN, Hilden, Germany) and were sequenced on the Illumina MiSeq platform (Illumina, San Diego, CA, USA) with v3 cartridges (600 cycles), following the manufacturer’s instructions. Negative controls were used in each round of sample processing steps.

Sequencing data were processed following a previously described pipeline^30^. The consensus sequences generated were classified using the Pangolin tool v.4.1.3. (https://github.com/cov-lineages/pangolin). All consensus sequences were deposited in the GISAID EpiCOV database. Information about the quality of the genomes and accession numbers is available in Supplementary Table S1.

A dataset (n = 456) composed of SARS-CoV-2 genomes available in the GISAID EpiCOV database was created to confirm the lineage classification and to contextualize the novel genomes generated in this study. This dataset comprised 262 genomes of SARS-CoV-2 that had been randomly selected and isolated from humans. These were classified as B.1.1.28, B.1.1.33, Zeta, Gamma, Delta and other lineages and variants. In addition, the dataset included all genome sequences from dogs and cats (85 and 109 genomes respectively) that were available at GISAID (i.e. available on December 22, 2021). This dataset was aligned using Minimap2 v.2.2.24 (Li, 2018), and maximum-likelihood phylogeny was inferred using IQ-tree v2.0.3, in conformity with the GTR + F + I + G4 model^31^. The Shimoidara–Hasegawa-like approximate likelihood ratio test (SH- aLRT) was used to measure phylogenetic uncertainty along with the tree branches^33^. Geneious prime 2023 v.1.2 was used to calculate the patristic genetic distance among the branches of the tree^34^. This paper was written according to the STROBE Statement.

### Statistical analysis

The test results and information on the animals that were the sources of the samples were tabulated in Microsoft Excel. The Statistical Package for the Social Sciences (SPSS, IBM, Armond, New York, USA) was used to analyze the data, and descriptive analyses were performed. The Mann–Whitney test was used to compare the RNA viral load between samples taken from oropharyngeal and rectal swabs, taken from asymptomatic and symptomatic animals.

### Supplementary Information

Supplementary Table 1.

Supplementary Table 2.

Supplementary Table 3.

## Data Availability

The datasets generated and/or analyzed during the current study are available from the corresponding author upon reasonable request.
