# Supplementary material for: Molecular detection and characterization of SARS-CoV-2 in cats and dogs of positive owners during the first COVID-19 wave in Brazil
Source: Sci Rep. 2023 Sep 2;13:14418. doi: 10.1038/s41598-023-41285-0 (PMC10475019; doi:10.1038/s41598-023-41285-0)
Supplement: Supplementary file 1 — Supplementary Table 1. [file 41598_2023_41285_MOESM1_ESM.docx]

**Supplementary Table 01.** Sequencing metrics and lineage classification of the genome samples.

| **Sequencing_**  **code** | **Original**  **Code** | **Lineage** | **Host** | **Collection_**  **date** | **number_of_**  **raw_reads** | **number_of_paired_**  **filtered_reads** | **number_of_unpaired_**  **filtered_reads** | **number_of_**  **mapped_reads** | **efficiency** | **average_**  **depth** | **coverage_**  **10x** | **coverage_**  **100x** | **coverage_**  **1000x** | **genome_**  **coverage** | **clade** | **Nextclade_**  **pango** |
| --- | --- | --- | --- | --- | --- | --- | --- | --- | --- | --- | --- | --- | --- | --- | --- | --- |
| SEQ_1196 | SP12F ARTEMIS | B.1.1.28 | cat | 2020-12-12 | 350584 | 334304 | 4816 | 321989 | 0.918436 | 1866.11 | 0.976992 | 0.806274 | 0.425777 | 0.978832 | 20B | B.1.1.28 |
| SEQ_1197 | CG006F | P.2 (Zeta) | cat | 2020-02-05 | 384138 | 366590 | 5336 | 353665 | 0.920672 | 1880.64 | 0.998194 | 0.948366 | 0.66291 | 0.998194 | 20B | P.2 |
| SEQ_1198 | CG008F | P.2 (Zeta) | cat | 2021-02-15 | 407568 | 381784 | 5914 | 363802 | 0.892617 | 1819.81 | 0.99806 | 0.971474 | 0.674347 | 0.998361 | 20B | P.2 |
| SEQ_1199 | SP25F RAVENA | P.1 (Gama) | cat | 2021-03-16 | 294774 | 278362 | 4669 | 269484 | 0.914205 | 1539.48 | 0.96288 | 0.860616 | 0.51249 | 0.965121 | 20J | P.1 |
| SEQ_1200 | SP28F SOL | P.1 (Gama) | cat | 2021-03-19 | 235554 | 219756 | 4098 | 213122 | 0.904769 | 1165.22 | 0.963148 | 0.85152 | 0.483162 | 0.965856 | 20J | P.1.14 |
| SEQ_1201 | CG027C | P.1.7 (Gama) | dog | 2021-07-13 | 259896 | 244704 | 4545 | 236370 | 0.909479 | 1369.35 | 0.969836 | 0.867605 | 0.49179 | 0.970337 | 20J | P.1.7 |
| SEQ_1202 | CG031F | P.1.7 (Gama) | cat | 2021-07-13 | 259746 | 243670 | 4658 | 235185 | 0.905442 | 1317.34 | 0.963984 | 0.881985 | 0.52162 | 0.966592 | 20J | P.1.7 |
| SEQ_1203 | CG028C | P.1 (Gama) | dog | 2021-07-13 | 264632 | 247690 | 4564 | 241887 | 0.91405 | 1344.57 | 0.675518 | 0.579005 | 0.434037 | 0.686486 | 20J | P.1.7 |
| SEQ_1204 | CG043F | P.1 (Gama) | cat | 2021-07-22 | 237926 | 224138 | 3778 | 218862 | 0.919874 | 1337.09 | 0.909708 | 0.747049 | 0.360432 | 0.917232 | 20J | P.1 |

| **Sequencing_**  **code** | **number_of_mapped_reads** | **efficiency** | **average_depth** | **coverage_10x** | **coverage_**  **100x** | **coverage_**  **1000x** | **genome_**  **coverage** | **clade** | **Nextclade_**  **pango** | **substitutions** | **aaSubstitutions** | **aaDeletions** |
| --- | --- | --- | --- | --- | --- | --- | --- | --- | --- | --- | --- | --- |
| SEQ_1196 | 321989 | 0.918436 | 1866.11 | 0.976992 | 0.806274 | 0.425777 | 0.978832 | 20B | B.1.1.28 | C3037T,A10323G,A12075G,G13127A,C14408T,C16859T,T20421C,A23403G,A23779G,G25088T,  C25460T,C25896T,G28221C,G28337A,G28881A,G28882A,G28883C | N:D22N,N:R203K,N:G204R,ORF1a:K3353R,ORF1a:N3937S,  ORF1a:G4288R,ORF1b:P314L,ORF1b:A1131V,ORF3a:A23V,  ORF8:E110Q,S:D614G,S:V1176F |  |
| SEQ_1197 | 353665 | 0.920672 | 1880.64 | 0.998194 | 0.948366 | 0.66291 | 0.998194 | 20B | P.2 | C3037T,C5284T,C5301T,T10667G,C11824T,C12053T,A13951G,C14408T,C16887T,A19020G,  T21937C,G22992A,G23012A,A23403G,G25088T,C28253T,A28254C,G28628T,G28881A,G28882A,  G28883C,G28975T,G29195T | N:A119S,N:R203K,N:G204R,N:M234I,N:A308S,ORF1a:A1679V,  ORF1a:L3468V,ORF1a:L3930F,ORF1b:I162V,ORF1b:P314L,  ORF8:I121L,S:S477N,S:E484K,S:D614G,S:V1176F |  |
| SEQ_1198 | 363802 | 0.892617 | 1819.81 | 0.99806 | 0.971474 | 0.674347 | 0.998361 | 20B | P.2 | C3037T,C4084T,A5368T,C6128T,T7033G,C10341G,T10667G,C11824T,C12053T,A12964G,  C14408T,G21123T,T21664C,G23012A,A23403G,G25088T,C27684T,C28253T,A28254C,G28628T,  G28881A,G28882A,G28883C,G28975T | N:A119S,N:R203K,N:G204R,N:M234I,ORF1a:R1701S,  ORF1a:L1955F,ORF1a:N2256K,ORF1a:P3359R,ORF1a:L3468V,  ORF1a:L3930F,ORF1b:P314L,ORF8:I121L,S:E484K,S:D614G,S:V1176F |  |
| SEQ_1199 | 269484 | 0.914205 | 1539.48 | 0.96288 | 0.860616 | 0.51249 | 0.965121 | 20J | P.1 | T733C,C843A,C2749T,C2773T,C3037T,C3828T,A5648C,A6319G,A6613G,C7851T,G11291A,T11296G,  C12318T,C12778T,C13860T,C14408T,G17259T,C21614T,C21621A,C21638T,G21974T,G23012A,  A23063T,A23403G,C23525T,C24642T,G25088T,G25252C,T26149C,G28167A,C28512G,A28877T,  G28878C,G28881A,G28882A,G28883C | N:P80R,N:R203K,N:G204R,ORF1a:P193H,ORF1a:S1188L,  ORF1a:K1795Q,ORF1a:A2529V,ORF1a:G3676S,ORF1a:F3677L,  ORF1a:S4018F,ORF1b:P314L,ORF1b:E1264D,ORF3a:S253P,  ORF8:E92K,ORF9b:Q77E,S:L18F,S:T20N,S:P26S,  S:D138Y,S:E484K,S:N501Y,S:D614G,S:H655Y,S:T1027I,S:V1176F |  |
| SEQ_1200 | 213122 | 0.904769 | 1165.22 | 0.963148 | 0.85152 | 0.483162 | 0.965856 | 20J | P.1.14 | T733C,C2749T,C3037T,C3828T,A5648C,A6319G,A6613G,C7851T,G9105A,G11291A,T11296G,  C12778T,C13426T,C13860T,C14408T,G17259T,A17961G,C21614T,C21621A,C21638T,G21974T,  G23012A,A23063T,C23380T,A23403G,C23525T,C24642T,G25088T,T26149C,G28167A,C28512G,  A28877T,G28878C,G28881A,G28882A,G28883C | N:P80R,N:R203K,N:G204R,ORF1a:S1188L,ORF1a:K1795Q,  ORF1a:A2529V,ORF1a:S2947N,ORF1a:G3676S,ORF1a:F3677L,  ORF1b:P314L,ORF1b:E1264D,ORF1b:I1498M,ORF3a:S253P,  ORF8:E92K,ORF9b:Q77E,S:L18F,S:T20N,S:P26S,  S:D138Y,S:E484K,S:N501Y,S:D614G,S:H655Y,S:T1027I,S:V1176F |  |
| SEQ_1201 | 236370 | 0.909479 | 1369.35 | 0.969836 | 0.867605 | 0.49179 | 0.970337 | 20J | P.1.7 | T733C,C1912T,C2749T,C3037T,C3828T,A5648C,A6319G,A6613G,G8618T,C9442T,G11291A,T11296G,  C12778T,C13860T,T14019C,C14408T,C16293T,G17259T,C21614T,C21621A,C21638T,G21974T,G23012A,  A23063T,A23403G,C23525T,C23604A,C24642T,G25088T,T26149C,G28167A,C28512G,A28877T,G28878C,  G28881A,G28882A,G28883C | N:P80R,N:R203K,N:G204R,ORF1a:S1188L,ORF1a:K1795Q,  ORF1a:A2785S,ORF1a:G3676S,ORF1a:F3677L,ORF1b:P314L,  ORF1b:E1264D,ORF3a:S253P,ORF8:E92K,ORF9b:Q77E,S:L18F,  S:T20N,S:P26S,S:D138Y,S:E484K,S:N501Y,S:D614G,S:H655Y,  S:P681H,S:T1027I,S:V1176F |  |
| SEQ_1202 | 235185 | 0.905442 | 1317.34 | 0.963984 | 0.881985 | 0.52162 | 0.966592 | 20J | P.1.7 | T733C,C1912T,C2749T,C3037T,C3828T,A5648C,A6319G,A6613G,G8618T,C9442T,G11291A,T11296G,  C12778T,C13860T,T14019C,C14408T,C16293T,G17259T,C21614T,C21621A,C21638T,G21974T,G23012A,  A23063T,A23403G,C23525T,C23604A,C24642T,G25088T,T26149C,G28167A,C28512G,A28877T,G28878C,  G28881A,G28882A,G28883C | N:P80R,N:R203K,N:G204R,ORF1a:S1188L,ORF1a:K1795Q,  ORF1a:A2785S,ORF1a:G3676S,ORF1a:F3677L,ORF1b:P314L,  ORF1b:E1264D,ORF3a:S253P,ORF8:E92K,ORF9b:Q77E,S:L18F,  S:T20N,S:P26S,S:D138Y,S:E484K,S:N501Y,  S:D614G,S:H655Y,S:P681H,S:T1027I,S:V1176F |  |
| SEQ_1203 | 241887 | 0.91405 | 1344.57 | 0.675518 | 0.579005 | 0.434037 | 0.686486 | 20J | P.1.7 | T733C,C1912T,C2094T,C3037T,C3828T,A5648C,A6319G,A6613G,C8240T,T8400C,C12778T,C13860T,  C14408T,C16293T,G17259T,C21614T,C21621A,C21638T,A22812C,G23012A,A23063T,A23403G,C23525T,  C24642T,G25088T,T26149C,G28167A,C28512G,G28541T,A28877T,G28878C,G28881A,G28882A,G28883C,  C29445T, | N:P80R,N:A90S,N:R203K,N:G204R,N:T391I,ORF1a:S610L,  ORF1a:S1188L,ORF1a:K1795Q,ORF1a:H2659Y,ORF1a:I2712T,  ORF1b:P314L,ORF1b:E1264D,ORF3a:S253P,ORF8:E92K,ORF9b:Q77E,  ORF9b:E86D,S:L18F,S:T20N,S:P26S,S:K417T,S:E484K,S:N501Y,S:D614G,  S:H655Y,S:T1027I,S:V1176F | ORF1a:S3675-,ORF1a:G3676-,ORF1a:F3677- |
| SEQ_1204 | 218862 | 0.919874 | 1337.09 | 0.909708 | 0.747049 | 0.360432 | 0.917232 | 20J | P.1 | C241T,T733C,C1912T,C2749T,C3828T,A5648C,A6319G,C9442T,G11291A,T11296G,C12778T,T14019C,C14408T,  T15561A,G17259T,G18242T,C21614T,C21621A,C21638T,A22812C,A23403G,C24642T,G25088T,G28167A,  A28877T,G28878C,G28881A,G28882A,G28883C | N:R203K,N:G204R,ORF1a:S1188L,ORF1a:K1795Q,ORF1a:G3676S,  ORF1a:F3677L,ORF1b:P314L,ORF1b:E1264D,ORF8:E92K,S:L18F,  S:T20N,S:P26S,S:K417T,S:D614G,S:T1027I,S:V1176F |  |
