# Supplementary material for: Molecular detection and characterization of SARS-CoV-2 in cats and dogs of positive owners during the first COVID-19 wave in Brazil
Source: Sci Rep. 2023 Sep 2;13:14418. doi: 10.1038/s41598-023-41285-0 (PMC10475019; doi:10.1038/s41598-023-41285-0)
Supplement: Supplementary file 3 — Supplementary Table 3. [file 41598_2023_41285_MOESM3_ESM.docx]

**Supplementary table 3.** Ct curves for N1 and N2 targets.

|  | Sample and species | Ct N1 | Ct N2 |
| --- | --- | --- | --- |
| 1 | Oropharyngeal swab from a dog | 34.75 | 31.2 |
| 2 | Oropharyngeal swab from a dog | 31.41 | 29.54 |
| 3 | Oropharyngeal swab from a dog | 33.20 | 31.00 |
| 4 | Oropharyngeal swab from a dog | 32.72 | 33.57 |
| 5 | Oropharyngeal swab from a dog | 35.47 | 34.28 |
| 6 | Oropharyngeal swab from a dog | 32.73 | 30.30 |
| 7 | Oropharyngeal swab from a cat | 27.81 | 27.15 |
| 8 | Oropharyngeal swab from a cat | 25.30 | 24.02 |
| 9 | Rectal swab from a dog | 36.42 | 36.6 |
| 10 | Oropharyngeal swab from a dog | 35.60 | 36.42 |
| 11 | Rectal swab from a dog | 34.97 | 35.45 |
| 12 | Oropharyngeal swab from a dog | 29.76 | 30.06 |
| 13 | Oropharyngeal swab from a dog | 27.63 | 28.00 |
| 14 | Oropharyngeal swab from a cat | 29.12 | 28.46 |
| 15 | Oropharyngeal swab from a cat* | 33.15 | 33.95 |
| 16 | Rectal swab from a cat* | 34.16 | 33.57 |
| 17 | Oropharyngeal swab from a cat | 29.73 | 31.17 |
| 18 | Oropharyngeal swab from a dog | 34.58 | 34.37 |
| 19 | Oropharyngeal swab from a cat | 28.99 | 29.15 |
| 20 | Oropharyngeal swab from a dog** | 33.25 | 30.78 |
| 21 | Rectal swab from a dog** | 35.90 | 35.45 |
| 22 | Oropharyngeal swab from a cat | 28.17 | 26.94 |
| 23 | Oropharyngeal swab from a cat | 25.12 | 26.25 |
| 24 | Oropharyngeal swab from a cat | 34.49 | 34.38 |
| 25 | Oropharyngeal swab from a cat | 31.65 | 33.46 |

* Same cat

** Same dog
